# The genomic landscape of *Pasteurella multocida* B:2 causing haemorrhagic septicaemia in Germany

**DOI:** 10.1186/s13567-026-01868-x

**Published:** 2026-10-29

**Authors:** Jörg Linde, Peter Kutzer, Jörg Rau, Reinhard Sting, Nina Kaestner, Annette Schliephake, Astrid König-Mozes, Tobias Eisenberg, Heinrich Neubauer, Johannes Solle, Rebecca Bonke, Anna Rettinger, Nicole Rudolph, Tibor Magyar, Kerstin Boll, Lisa Schneider-Bühl, Lisa D. Sprague

**Affiliations:** 1 Friedrich-Loeffler Institut, Institut für Bakterielle Infektionen und Zoonosen, Jena, Germany; 2 Landeslabor Berlin-Brandenburg, Institut für Lebensmittel, Arzneimittel, Tierseuchen und Umwelt, Frankfurt (Oder), Germany; 3 Chemisches und Veterinäruntersuchungsamt Stuttgart, Fellbach, Germany; 4 Bayerisches Landesamt für Gesundheit und Lebensmittelsicherheit, Erlangen, Germany; 5 Landesamt für Verbraucherschutz Sachsen-Anhalt, Halle (Saale), Germany; 6 Landesuntersuchungsamt Rheinland-Pfalz, Koblenz, Germany; 7 Landesbetrieb Hessisches Landeslabor (LHL), Gießen, Germany; 8 Landesuntersuchungsanstalt für das Gesundheits- und Veterinärwesen Sachsen, Leipzig, Germany; 9 HUN-REN Veterinary Medical Research Institute, Budapest, Hungary

**Keywords:** *Pasteurella multocida*, haemorrhagic septicaemia, sequencing, SNP, cgMLST, MLST, genotype

## Abstract

Haemorrhagic septicaemia (HS) is a peracute to acute, highly fatal disease caused by *Pasteurella* (*P.*) *multocida* serogroups B:2 and E:2 affecting cattle and buffaloes. HS in cattle and buffaloes necessitates mandatory reporting to the World Organisation for Animal Health (WOAH; formerly OIE) due to its significant socio-economic impact on livestock production and international trade. In other ungulate species infection with *P. multocida* B:2 (iPmB:2) presents with a similar clinical picture. A series of HS and iPmB:2 outbreaks between 2010 and 2024 in Germany required a detailed investigation into the spatiotemporal dynamics and molecular characteristics of the circulating strains. The ability to specifically identify and analyse circulating serovars is crucial for confirming outbreaks and implementing appropriate control measures. A total of 221 *Pasteurella multocida* capsular type B:2 isolates associated with HS/iPmB:2 outbreaks was collected between 2010 and 2024 from dead livestock and wildlife in Germany in the context of veterinary practice and subsequently analysed using whole-genome sequencing. Isolates were characterised by capsular and lipopolysaccharide PCR-based typing, multilocus sequence typing, and high-resolution genomic comparison. Four high-resolution-typing approaches were evaluated and compared with epidemiologically defined outbreaks to identify the most suitable method for outbreak investigation. Single nucleotide polymorphism (SNP)-based analysis showed the highest discriminatory power and best agreement with known outbreak data, outperforming conventional multilocus sequence typing (MLST) and core-genome multilocus sequence typing (cgMLST) approaches. Genotyping identified eight major genetic clusters of closely related strains across multiple federal states and host species. Several outbreaks were linked by highly similar or identical genomes, suggesting both local persistence and wider geographic spread of the pathogen within Germany.

**Supplementary Information:**

The online version contains supplementary material available at https://doi.org/10.1186/s13567-026-01868-x.

## Introduction

*Pasteurella* (*P.*) *multocida* is a Gram-negative, non-motile, facultative anaerobic, pleomorphic coccobacillus within the *Pasteurellaceae* family. It possesses a remarkable capacity to colonise a wide array of animal species, ranging from domestic livestock to companion animals, poultry and wildlife, often residing asymptomatically in the mucous membranes of the upper respiratory and intestinal tract as a commensal organism [1]. *P. multocida* exhibits considerable genetic diversity and isolates are classified into five capsular serogroups (A, B, D, E and F) and 16 somatic serovars based on the presence of distinct capsule and somatic antigens [2]. The capsular serogroups are largely associated with specific disease manifestations and host predilections; symptoms associated with *P. multocida* infection in wild and domestic animals can range from asymptomatic or mild chronic upper respiratory inflammation to acute, regularly fatal pneumonic and/or disseminated disease. The most common diseases associated with *P. multocida* are atrophic rhinitis and pneumonia in pigs, pleuropneumonia and mastitis in sheep, fowl cholera in poultry, snuffles in rabbits and shipping fever in cattle.

Haemorrhagic septicaemia (HS) is a peracute to acute, highly fatal disease caused by *P. multocida* serogroups B:2 and E:2 (Carter and Heddleston system) affecting cattle and buffaloes [3]. Recognised as a notifiable disease by the World Organisation for Animal Health (WOAH; formerly OIE), HS in cattle and buffaloes necessitates mandatory reporting due to its significant socio-economic impact on livestock production and international trade. In other ungulate species (e.g. red deer, fallow deer, roe deer, elk, mouflon, horse, swine and wild boar) infection with *P. multocida* B:2 (iPmB:2) presents with a similar clinical picture. Carnivores can be asymptomatic carriers [3–5].

In the Federal Republic of Germany (BRD), the notification and economically burdensome [disease] control obligation of HS was abolished in 1969, as the disease was no longer detected and most likely only occurred sporadically. Voluntary reporting to the animal disease information system (TSN) was possible, however the data are not informative. The former German Democratic Republic (GDR) had its own legislation regarding the notification of HS which was abolished in 1991 after the unification of Germany in October 1990. No cases of HS were reported between 1949 and 1991. As of March 10, 2026, HS is again a notifiable disease in Germany.

A concerning series of HS outbreaks in Germany between 2010 and 2024, previously described by [4, 5], necessitated a detailed investigation into the spatiotemporal dynamics and molecular characteristics of the circulating strains [in Germany] in order to understand the source, spread, and characteristics of the causative agent. The ability to specifically identify and analyse HS-associated serovars is crucial for confirming outbreaks and implementing appropriate control measures. This manuscript aims to fill a critical gap by leveraging advanced genomic techniques to develop a high-resolution typing strategy based on a German HS hybrid genome for German *P. multocida* B:2 strains. In doing so, we provide a comprehensive geographic map of genotype distribution, as well as the presence of the pathogen across affected regions.

## Materials and methods

### Bacterial cultivation, bacterial conservation and DNA extraction

A total of 221 *P. multocida* B:2 isolates collected and identified between 2010 and 2024 by the responsible state laboratories was sent to and confirmed anew at the Institute of Bacterial Zoonoses at the Friedrich-Loeffler-Institute in Jena (IBIZ-FLI). As detailed in Additional file 3, we analysed isolates from cattle (*n* = 136), wild boar (*n* = 32), pig (pig, piglet, domestic pig, Göttinger Minipig, babirusa; *n* = 21), fallow deer (*n* = 13), water buffalo (*n* = 8), deer (*n* = 5), wolf (*n* = 5) and horse (*n* = 1). The isolates were shipped on AMIES swabs with charcoal (Sarstedt; Nümbrecht, Germany) and cultivated on Columbia blood agar containing 5% sheep blood (Thermo Fisher Scientific; Darmstadt, Germany) for 24 h at 37 °C. A single colony was transferred into 10 mL LB-medium (Thermo Fisher Scientific; Darmstadt, Germany) and incubated at 37 °C in a shaking incubator for 24 h. Prior to genomic DNA extraction using the QIAGEN Genomic-tip 20/G and Genomic DNA Buffer Set (Qiagen, Hilden, Germany) according to the manufacturer’s instructions, two cryovials per isolate were stored at − 80 °C using the Microbank^R^ Preservation System (Pro-Lab Diagnostics; VWR, Germany). Moreover, in order to exclude cultivation bias, five randomly picked isolates (24Y00130, 24Y00039, 24Y00111, 17Y0497 and 24Y00005) were serially subcultured over 20 passages and a respective DNA sample was sequenced after every 5th subculture.

### PCR

At the IBIZ-FLI all isolates were submitted to *P. multocida* multiplex capsular PCR typing [6] and *P. multocida* multiplex LPS PCR typing [7] as described.

### Genome sequencing of *Pasteurella multocida* B:2 isolates

DNA purity was assessed with a Colibri spectrophotometer (Thermo Fisher Scientific; Schwerte, Germany) and quantified using a Qubit™ 3 Fluorometer with the Qubit™ double-stranded DNA (dsDNA) high-sensitivity (HS) Assay Kit (Invitrogen™; Schwerte, Germany). The paired-end genome sequencing library was generated with the Nextera XT DNA Library Preparation Kit (Illumina Inc., San Diego, CA, USA) followed by whole genome sequencing using an Illumina MiSeq instrument to generate reads 300 bp in length according to the manufacturer’s instructions. With the aim of constructing a complete and closed reference genome for high resolution SNP typing, isolate 24Y00006 was selected from the predominant sequence type (ST64) within the German *P. multocida* B:2 isolate collection and subjected to additional long-read sequencing. The use of a reference from the population under study provided a closely related reference for read mapping and SNP calling rather than relying on a more distantly related RefSeq genome. Isolate 24Y00006 was sequenced with a MinION Mk1B device (Oxford Nanopore Technologies; Oxford, UK) using the ONT Native Barcoding Kit 24 V14 (REF: SQK-NBD114.24) according to the manufacturer’s instructions.

### Raw data analysis

The paired-end data were analysed using the WGSBAC pipeline [8] (v2.2.3) which included quality control of raw sequencing data with FastQC (v0.11.5). Raw coverage was calculated by multiplying the number of reads by their average length and dividing them by the genome size. Shovill (v1.0.4), which incorporates the SPAdes genome assembler (v3.14.0), was used to assemble the raw reads. Assembly quality was assessed by using QUAST (v5.0.2). To evaluate the continuity of assemblies, we used the N50 metric, which reflects the minimum contig length needed to cover 50% of the genome. To detect potential inter-species contamination, reads and assemblies were classified using Kraken 2 (v2.0.7 beta) with the MiniKraken (v2.) database. To detect genetic markers for antimicrobial resistance, AMRFinderPlus (v3.11) was applied. For detection of genes coding for virulence factors and plasmid replicons ABRicate (v1.0.1) was used with the Virulence Factor and PlasmidFinder database, respectively.

### Genotyping

For prediction of the capsule type (Carter) and the lipopolysaccharide (LPS) group, ABRicate was used with reference sequences downloaded from the *Pasteurella multocida* Sequence Database [9]. *In silico* capsule and LPS typing was performed in parallel with PCR typing to evaluate if these features could be reliably determined directly from the genome assemblies. Multilocus sequence typing (MLST) was performed with the software mlst [10] (v2.23.0) based on the Multi-host MLST Scheme [11] and the RIRDC MLST scheme [12] both downloaded from the PubMLST database (accessed May 2025).

In order to evaluate which genotyping approach performed best for *P. multocida* B:2 isolate typing in Germany, we tested four complementary methods and assessed their performance against epidemiologically defined outbreak data (Additional file 3 and Additional file 5).

First, SNP-based typing was performed using the hybrid assembly (long and short reads, i.e. ONT and Illumina) of isolate 24Y00006 as the reference genome (24Y00006ref). Reads were mapped and SNP-called with Snippy [13] (4.6.0), generating an alignment of genomic positions shared across all isolates (core-genome alignment). SNPs identified within this shared sequence, including coding and non-coding regions, are hereafter referred to as core-genome SNPs (cgSNPs). To account for potential recombination, the alignment was additionally processed with Gubbins [14] (v3.4), generating a recombination-filtered SNP dataset. For both the unfiltered and filtered approaches, pairwise cgSNP distances were calculated using snp-dists [15] (v0.7.0) and dendrograms were generated from the resulting distance matrices.

Next, we applied core-genome multilocus sequence typing (cgMLST), which compares isolates based on allelic differences across a defined set of conserved genes. In one setting, allele calling was based on a publicly available cgMLST scheme comprising 1238 loci, converted from BigsDB [16] for analysis with ChewBBACA [17] (v3.3.4). In addition, we developed an *ad hoc* cgMLST, i.e. a German-specific scheme (cgMLST_Ger) from the assemblies of 221 isolates, resulting in 1999 loci. The *ad hoc* scheme was generated solely to evaluate a dataset-specific cgMLST approach. For both schemes, the ChewBBACA-derived allele profiles were processed with the Chewiesnake [18] pipeline to assign alleles and to compute pairwise allele distance matrices. Finally, distance matrices from all four approaches (unfiltered SNPs, recombination-filtered SNPs, public cgMLST, and *ad hoc* cgMLST) were used for hierarchical clustering. Clusters were defined at thresholds of 0, 1, 5, 10, 20, and 50 SNPs or alleles.

For each genotyping approach and clustering threshold, discriminatory power was assessed using Simpson’s Index of Diversity [19], with values closer to 1 indicating greater discriminatory power. Agreement between the genomic clustering and the eight epidemiologically defined outbreaks was assessed using the Adjusted Rand Index (ARI) [20], where an ARI of 1 indicates perfect agreement and an ARI of 0 indicates agreement no better than expected by chance.

The eight outbreaks used as epidemiological reference grouping for comparison included 75 isolates and were determined as follows: The primary outbreak of *P. multocida* B:2 in 2010, initially affecting wild fallow deer (*Dama dama*), originated within the Fläming Heath, a range of hills covering an area of approx. 4300 square kilometres, situated in the two federal states of Brandenburg and Saxony-Anhalt. The competent authorities therefore, defined a survey area centred on the index case, which encompassed several neighbouring districts; over a period of eight months (July 2010 to February 2011), the latter were subject to close monitoring including farm animals, wildlife and the environment [4]. In December 2010, *P. multocida* B:2 was recovered from a roe deer and in February 2011, the last *P. multocida* B:2 strains were isolated from clinically healthy fenced fallow deer of a previously affected herd. A later outbreak in this area in 2013, was caused by a different genotype. Exact spatiotemporal metadata obtained from several successive closely monitored outbreaks in Bavaria (2017), Baden-Wuerttemberg (2019) and Saxony (2019, 2021 and 2022; personal communication Dr. M. Schmidt) were used for validation of the typing system (Additional file 3). No fixed temporal cut-off was applied; the temporal association was assessed on a case-by-case approach based on the available epidemiological information for each outbreak, provided by the participating veterinarians, state laboratories and from published literature [4, 5].

## Results

### Genomic characterisation of *Pasteurella multocida* B:2 genomes from Germany

In this study, 221 *P. multocida* B:2 isolates from Germany were submitted to short-read sequencing. Each genome yielded at least 0.6 million reads, providing a minimum coverage of 69-fold (Additional file 4). At least 74% of the sequenced nucleotides per isolate reached Q30 (average 86%). The assembly process produced between 19 and 49 contigs, with N50 values ranging from 85.3 to 596.8 kbp. At least 98% of raw reads per isolate were classified as *P. multocida*. GC content ranged between 40.28% and 40.38%. The hybrid assembly of isolate 24Y00006 (i.e. 24Y00006ref) consisted of one contig and 2.3 million bp.

None of the genomes carried plasmid replicons or known genetic markers for antimicrobial resistance (data not shown). Screening against the Virulence Factor Database identified *lpx*A, *lpx*C, *rfa*D in all isolates These genes encode proteins involved in lipopolysaccharide (LPS) synthesis in Gram-negative bacteria.

Based on the genome assemblies, LPS group 2 (Additional file 4) was predicted for all isolates. Moreover, all isolates carried capsule type B (Additional file 4). According to the RIRDC MLST scheme, all isolates were assigned to sequence type (ST) 122 (Additional file 5). Using the multihost MLST scheme, 207 isolates were classified as ST 64, whereas 14 isolates belonged to ST 61. These two sequence types differ only at the *aro*A locus, while the remaining six loci are identical.

### Evaluation of typing approaches

Four typing approaches were compared, i.e. SNP typing (SNP), SNP typing with Gubbins filter for recombination (SNP_Gub), cgMLST using the publicly available scheme (cgMLST), and cgMLST using the German-specific *ad hoc* scheme (cgMLST_Ger) (Additional file 6). These approaches were evaluated across hierarchical clustering thresholds of 0, 1, 5, 10, 20 and 50 SNPs or alleles. For each method-threshold combination, the number of partitions, Simpson’s Index of Diversity, and agreement with the defined outbreaks using the Adjusted Rand Index (ARI) were determined. SNP-based methods captured the outbreak structure most effectively (Table 1). At threshold 1, both SNP and SNP_Gub produced 38 partitions, with high diversity (Simpson’s index = 0.93) and moderate agreement with the outbreak definition (ARI = 0.73). Increasing the threshold to 10 reduced the number of partitions to eight, with a Simpson’s Index of Diversity of 0.87, while increasing the ARI to 0.84, indicating the highest degree of concordance with the defined outbreaks. Higher thresholds (20 and 50) merged clusters excessively, dropping the ARI below 0.2 and eliminating discriminatory power (Simpson’s index ≤ 0.35). Filtering for recombination had no effect on clustering results, as SNP-based clusters before and after recombination filtering were identical. In detail, we quantified SNP density in 1 kb windows across the core genome. The majority of windows (91%) contained a single SNP, while only ~9% contained ≥2 SNPs, with a maximum of 4 SNPs per window.

Though cgMLST approaches detected at least 95% of loci, they showed limited ability to resolve outbreaks. The pubMLST scheme consistently produced only a single partition at clustering thresholds 5 alleles and above, resulting in zero diversity and no agreement with epidemiological groupings. The German-specific cgMLST scheme performed better at lower thresholds. At threshold 1, it generated 54 partitions with high diversity (Simpson’s index = 0.91), but the ARI reached only 0.69, suggesting that the clusters did not align well with actual outbreaks. Overall, SNP-based methods outperformed cgMLST by offering both finer resolution and stronger epidemiological concordance, particularly at intermediate clustering thresholds. To investigate the lower resolution of cgMLST compared to SNP-based typing, we first quantified the proportion of SNPs located in intergenic regions. Out of 160 unique core-genome SNPs, 22 (14%) were intergenic. When focusing on cgMLST loci, 304 SNPs (including non-unique events) were compressed into 259 allelic differences (compression factor 1.17). This corresponds to a reduction of fine-scale variation by ~15%, consistent with the broader clustering and lower discriminatory power observed in cgMLST compared to SNP analysis.

Table 1 shown are the numbers of resulting partitions and the Simpson’s Index of Diversity, which reflect how well a typing scheme distinguishes strains (higher values indicate greater discriminatory power). The Adjusted Rand Index (ARI) values quantify how closely each clustering method matches eight outbreaks comprising 75 isolates; an ARI of 1 indicates perfect agreement, whereas 0 indicates no better than random.

**Table 1:** **Comparison of typing approaches**

Approach	Threshold	Partitions	Simpson’s	ARI
SNP				
	1	38	0.93	0.73
	5	22	0.93	0.78
	10	8	0.87	0.84
	20	2	0.19	0.35
	50	1	0.00	0.00
SNP_Gub				
	1	38	0.93	0.73
	5	22	0.93	0.78
	10	8	0.87	0.84
	20	2	0.19	0.35
	50	1	0.00	0.00
cgMLSTpubMLST				
	1	14	0.49	0.29
	5	1	0.00	0.00
	10	1	0.00	0.00
	20	1	0.00	0.00
	50	1	0.00	0.00
cgMLSTGer				
	1	54	0.91	0.69
	5	4	0.23	0.29
	10	1	0.00	0.00
	20	1	0.00	0.00
	50	1	0.00	0.00

### Genomic signatures of epidemiologically defined outbreaks

**Figure 1:**
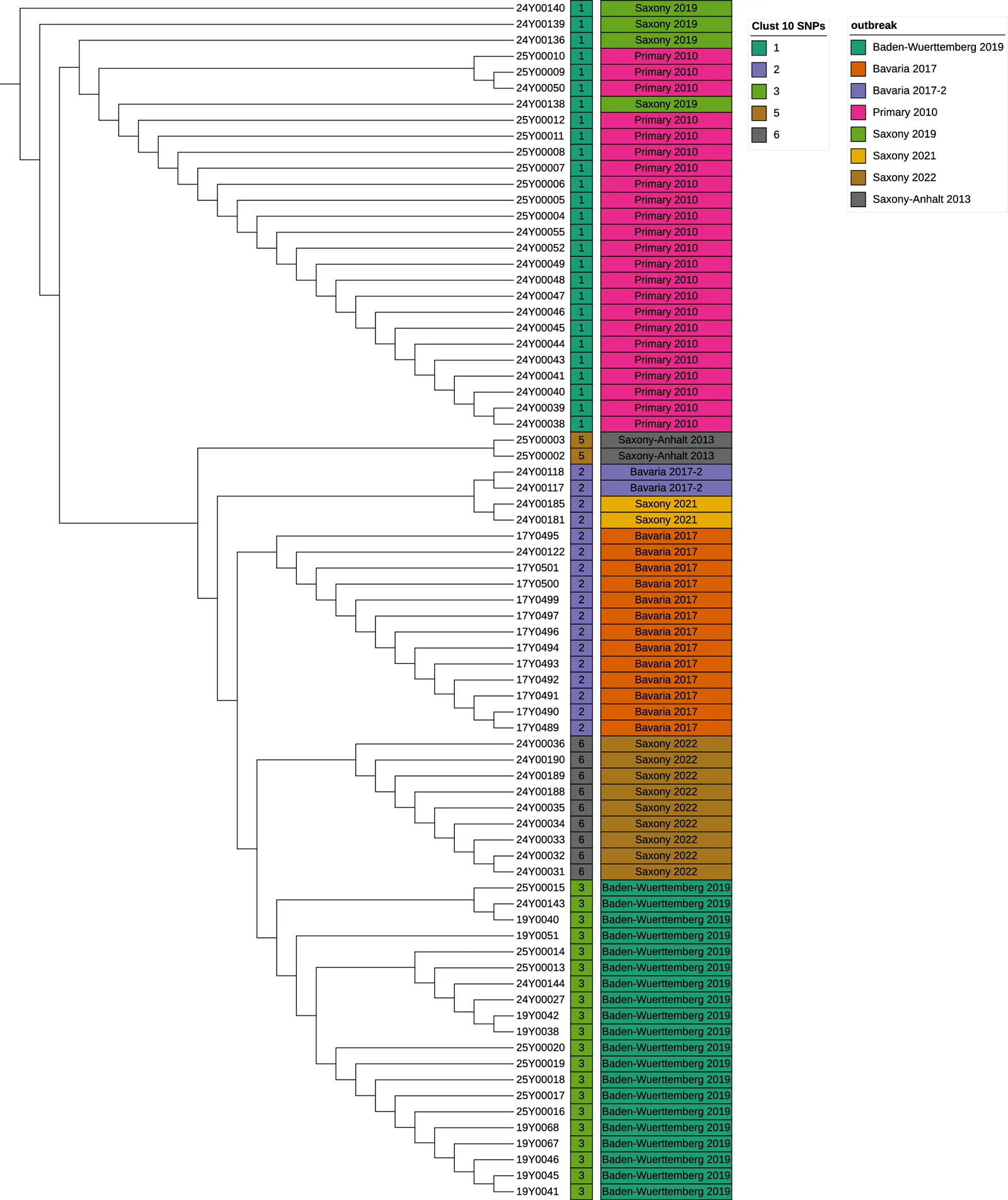
***Pasteurella multocida* B:2 outbreaks in Germany.** SNP based dendrogram from hierarchical clustering of the 75 isolates used to design the German-centric typing scheme. Branch lengths in the iTOL visualisation are not scaled; terminal branches are displayed with sufficient spacing to accommodate isolate labels and epidemiological annotations. Cluster number and outbreaks are based on the data from all 221 German isolates analysed (Additional file 1).

In 2010, the first upsurge of *P. multocida* B:2 occurred in the federal states of Brandenburg and Saxony-Anhalt in the Fläming Heath. By 2013, hundreds of animals—including pigs, cattle, roe deer, and fallow deer—had been affected. All twenty-three isolates from this (primary) outbreak fall within cluster 1 at the 10-cgSNP threshold, while two isolates (25Y00002, 25Y00003) from the same area but isolated in 2013 belong to cluster 5 (Figure 1; Additional file 3) indicative of an independent outbreak. At the more stringent threshold of 0-cgSNPs, 20 isolates from both federal states group into subcluster 1, demonstrating genomic identity. This subcluster also includes 14 isolates collected between 2011 and 2023 across northern and central Germany.

Two outbreaks occurred in 2017 in Bavaria. Fifteen isolates were analysed, originating from ten fenced wild boar, three fenced deer, and two grazing cattle. At the 10-cgSNP threshold, these isolates fall within cluster 2. Eleven were collected in farm A and two in farm B (25 km distance; municipality Bayreuth; Bavaria 2017).

The second outbreak occurred in the municipality of Amberg-Sulzbach (53 km from farm A; municipality Bayreuth; Bavaria 2017-2) affecting two wild boars. At the 0-cgSNP threshold, fourteen isolates group into (multihost) subcluster ST64, indicating they are genomically identical, while one isolate (24Y00122) from farm B (municipality Bayreuth) belongs to subcluster ST61. Beyond these outbreaks, cluster 2 at 10-cgSNPs also comprises 33 additional isolates, including isolates 24Y00181 and 24Y00185 from a 2022 outbreak in Saxony.

In 2019, an outbreak occurred in Baden-Wuerttemberg, centred around the municipality of Calw. Twenty cattle-derived isolates were analysed. All isolates fall within cluster 3 at the 10-cgSNP threshold and form four subclusters with 0-cgSNP difference (subclusters 3, 6, 11, and 22 at 0-cgSNPs). Three subclusters consist exclusively of isolates from Baden-Wuerttemberg collected in 2019, whereas subcluster 11 also includes three outbreak isolates together with isolate 24Y00090, isolated in 2016 from a wild boar in Bad Fallingbostel (Lower Saxony).

In 2018/2019, an outbreak was reported in the municipalities of Bautzen and Görlitz in Saxony, bordering directly on Poland. All four isolates fall within cluster 1 at the 10-cgSNP threshold, the same cluster that contained 93 genomes from seven federal states, including those from the primary 2010 outbreak in Brandenburg and Saxony-Anhalt. Notably, isolate 24Y00138, collected in February 2019 in the municipality of Bautzen, shows no cgSNP differences when compared to 20 isolates from the 2010 outbreak, as all belong to cluster 1 at the 0-cgSNP threshold.

In 2021, another outbreak occurred in Saxony, centred around the Vogtlandkreis and a further one in 2022, in the Erzgebirgskreis. Eleven cattle-derived isolates were analysed, falling into two clusters at the 10-cgSNP threshold: cluster 2 (*n* = 2) and cluster 6 (*n* = 9). Eight of the cluster 6 isolates share 0-cgSNPs and form subcluster 5 at 0-cgSNPs. In addition to the Saxonian isolates, cluster 6 at 10-cgSNPs also contains three isolates from Brandenburg collected in 2021 and 2022. The two cluster 2 isolates from the Vogtlandkreis, Saxony likewise share 0 cgSNPs. In total, cluster 2 comprises 48 isolates from six federal states.

### The genomic landscape of *Pasteurella multocida* B:2 in Germany

**Figure 2:**
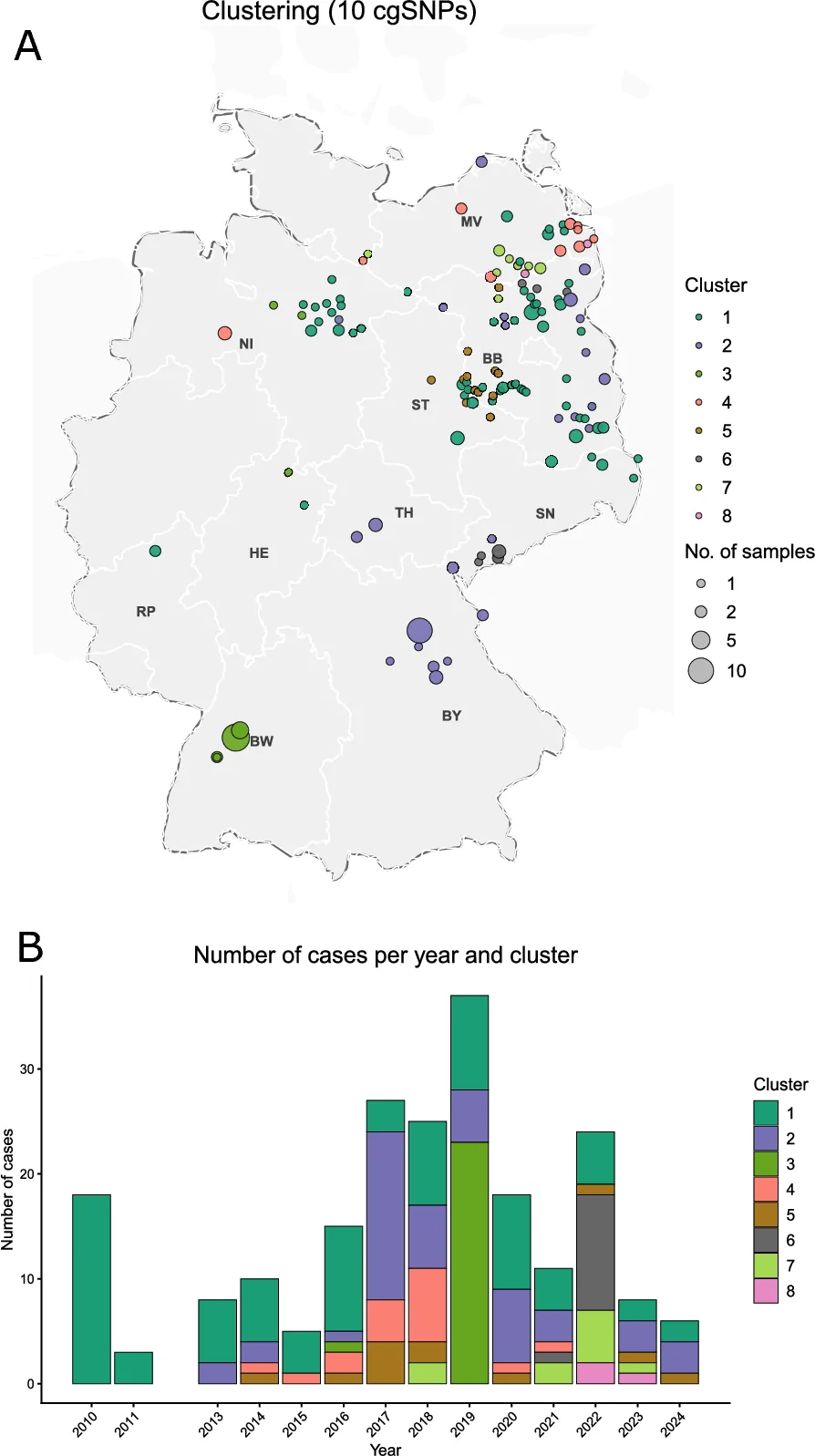
**Geographic location and temporal dynamics of 221 German** ***Pasteurella multocida*** **B:2 isolates based on cgSNP analysis. (A)** Geographic distribution of isolates across Germany. Each location is coloured according to its 10-cgSNP cluster, visualising the spatial spread and distribution of genetically related isolates. Federal states are labelled as follows: BW: Baden-Wuerttemberg; BY, Bavaria; BB: Brandenburg; HE: Hesse; NI: Lower Saxony; MV: Mecklenburg-Western Pomerania; RP: Rhineland-Palatinate; SN: Saxony; ST: Saxony-Anhalt; TH, Thuringia. **B** Temporal distribution of isolates by year of isolation. Bars are coloured based on 10-cgSNP clusters, illustrating the persistence and emergence of clusters over time.

In addition to the outbreak scenarios described above, we examined the overall genomic population structure of all 221 *P. multocida* B:2 isolates using cgSNP typing at a 10-cgSNP threshold (unless stated otherwise). This grouped the isolates into eight clusters containing at least three isolates (Figure 2, Additional file 1, Additional file 3). Cluster 1 emerged as the largest group, comprising 93 isolates collected between 2010 and 2023. These isolates originated from seven northern and central federal states, while no isolates from Bavaria, Baden-Wuerttemberg, or Thuringia appeared in this cluster (Figure 2, Additional file 1). At a 0-cgSNP threshold, cluster 1 further splits into 13 subclusters (Additional file 2). Subcluster 1, for example, contains 35 isolates collected over a 13-year period from six different states. Several subclusters show spatial and temporal containment, such as subcluster 13, which includes four isolates obtained in 2020 in the Oberhavel municipality, and subcluster 17, which groups three isolates collected between 2016 (24Y00082; 24Y00083) and 2018 at 17 km distance (24Y00016) also within the municipality of Potsdam Mittelmark.

Cluster 2 comprises 48 isolates collected between 2013 and 2024 from six German federal states (Figure 2). In total, cluster 2 contains twelve subclusters. Subcluster 2, defined at 0-cgSNPs, includes twelve isolates collected in 2017 in Bavaria and three isolates from Brandenburg, including isolate 24Y00085, which was collected from a wild boar in 2016 (Additional file 2). Subcluster 7 consists of five isolates acquired in 2020 in the Franconia region: four from cattle and one from a babirusa (pig). A further isolate, 24Y00117, obtained from a wild boar in 2017, also belongs to this subcluster.

Cluster 3 comprises 24 isolates, of which 21 were collected in Baden-Wuerttemberg in the municipality of Calw. All isolates in cluster 3 were collected in 2019 with the exception of a single isolate from 2016. The majority of isolates in this cluster originated from cattle, with three exceptions: wild boar isolates from Lower Saxony and Hesse. Twenty isolates from the outbreak in Baden-Wuerttemberg in 2019 are part of the cluster (see below). Cluster 3 contains four subclusters of isolates sharing 0-cgSNPs, including subcluster 3, which groups with ten isolates from Calw.

Seventeen isolates from northern Germany are grouped in Cluster 4. Nine of these, isolated in Mecklenburg-Western Pomerania between 2015 and 2018, share 0-cgSNPs and form subcluster 4. Subcluster 18 includes three wolf-derived isolates collected in Barnstorf, Lower Saxony, while subcluster 34 groups two cattle-derived isolates from the municipality Ostprignitz Ruppin in Brandenburg.

Cluster 5 contains 14 isolates from Saxony-Anhalt and Brandenburg, isolated between 2013 and 2023 from various host species. Six of these isolates group into two subclusters at 0-cgSNPs: subcluster 12 (four isolates) and subcluster 32 (two isolates). The remaining isolates in cluster 5 do not group at the 0-cgSNP threshold, indicating greater genetic diversity within this cluster.

In total, cluster 6 includes 12 *P. multocida* B:2 isolates—nine from Saxony and three from Brandenburg. All isolates were obtained in 2022 with one exception isolated in 2021. Eight isolates sharing 0-cgSNPs, all collected from cattle in Saxony in 2022, form one subcluster. A second subcluster consists of two isolates from Brandenburg, isolated from fallow deer and cattle, also show complete genomic identity (0 cgSNPs).

Cluster 7 groups ten isolates: eight from cattle, one from fallow deer, and one from wild boar. Nine were collected between 2018 and 2023 in Mecklenburg-Western Pomerania and one in Brandenburg in 2023. Subcluster 15 includes three isolates from the Mecklenburgische Seenplatte: 24Y00192 from cattle and two additional isolates from cattle (24Y00201) and fallow deer (24Y00026) collected at approximately 19 km distance. A second subcluster contains three cattle-derived isolates with identical genomes.

The final cluster 8, based on a 10-cgSNP threshold, includes three completely identical isolates. These isolates, which also cluster at 0-cgSNPs, were isolated in Mecklenburg-Western Pomerania from cattle (*n* = 2) and a fallow deer in 2022.

### Comparison to European sequencing data

The search in the Sequence Read Archive resulted in raw sequencing data of 49 samples [5, 21], isolated in seven European countries from twelve different host species (Additional file 7). LPS analysis detected five different LPS groups, where the majority of isolates belong to LPS group 3 (*n* = 21) causing among other shipping fever [22] and LPS group 6 associated with swine respiratory disease (*n* = 15). Five isolates from Hungary, isolated between 2013 and 2018 from cattle (*n* = 2), fallow deer, goat and pig belong to LPS group 2. SNP analysis grouped isolates pm4586 (ID: SRR13148986) and pm3987 (SRR13148935), isolated in 2020 and 2019 from fallow deer and cattle, respectively, in Hungary, with all 35 isolates of subcluster 1 in Germany at 0 cgSNPs, indicating a relationship, previously highlighted by [5]. Notably, subcluster 1 includes 20 isolates from the primary outbreak. Isolates P57 (ID: SRR13148936) isolated in 2013 from a pig in Hungary, clustered at 0-cgSNPs with four isolates (24Y00067, 24Y00089, 25Y00002, 25Y00003) from Brandenburg and Saxony-Anhalt.

## Discussion

This study presents a comprehensive analysis of 221 German HS *P. multocida* B:2 and infection with *P. multocida* B:2 (iPmB:2) isolates with next-generation sequencing (NGS)-based genotyping approaches using a German HS hybrid genome and isolates from German outbreaks for evaluation and high resolution using 10-cgSNP and 0-cgSNP. Our findings provide new insights into the molecular epidemiology, phylogeography, and outbreak dynamics of this important pathogen, especially within the context of German wild-life and livestock production systems.

NGS-derived data confirmed that all examined isolates belong to capsular type B (CAP-B), in agreement with traditional PCR-based capsular typing. Likewise, lipopolysaccharide (LPS) genotyping using in silico tools such as ABRicate yielded results consistent with PCR-based LPS typing.

Multilocus sequence typing (MLST) was evaluated using both the legacy RIRDC scheme and the more recent multihost MLST approach. All isolates were assigned to ST122 under the RIRDC scheme, providing no discriminatory resolution. Using the multihost MLST scheme, the majority of isolates was assigned to ST64, while a smaller proportion belonged to ST61. These two sequence types differed only at the *aro*A locus and shared the same virulence marker profiles. Neither ST64 or ST61 showed an apparent host preference in our dataset, with ST 61 isolates recovered from cattle, water buffalo, pigs, wild boar and wolf. These results are in accordance with previously observed findings [5] and underscore the limited resolution capacity of conventional MLST at distinguishing closely related *P. multocida* B:2 isolates.

In order to obtain a better resolution of genetic relationships among *P. multocida* B:2 isolates, we evaluated four high-resolution typing methods: (i) cgSNP-based typing, (ii) cgSNP typing with recombination filtering using Gubbins (SNP_Gub), (iii) core genome MLST (cgMLST) using the pubMLST scheme, and (iv) cgMLST based on a customised German-specific scheme (cgMLST_Ger). While cgMLST approaches offered a modest improvement over traditional MLST in resolving genetic variation, they still exhibited limited discriminatory power within this clonal lineage. Whole-genome SNP typing provided the highest resolution, clearly distinguishing isolates within and across epidemiologically defined outbreaks (Figure 1). This reduced resolution of cgMLST can be explained by two factors: first, cgSNPs were located in intergenic regions and therefore not captured by cgMLST; second, cgMLST compressed several cgSNPs within loci into one allelic difference.

Our analysis further showed that hierarchical clustering based on a threshold of 10 cgSNPs provided the best fit with the defined outbreaks in the German isolate collection analysed in this study (Additional file 1). However, this threshold has not been independently validated in a separate dataset and should therefore not be considered a universal threshold for *P. multocida* B:2. Further validation using independent and geographically diverse isolate collections is required to assess its broader applicability. Remarkably, the inclusion or exclusion of recombination events, assessed via Gubbins filtering, had no impact on the clustering outcome. In addition to the observed lack of a recombination signal, the cgSNPs also did not form genomic clusters (their genomic locations are scattered), which might confound SNP-based hierarchical clustering. This could reflect the high genetic similarity and clonal nature of the German B:2 isolate population analysed in this study. However, recombination could become a more significant confounding factor in studies involving geographically or genomically more diverse *P. multocida* populations, where horizontal gene transfer may play a larger role in shaping genomic diversity. The influence of transport medium and time, laboratory SOPs, sequencing platform and sequencing chemistry, but also experience of bioinformaticians has to be assessed in future studies.

Detailed analysis of outbreak-associated isolates revealed high genomic similarity within distinct outbreaks, with most isolates clustering into single clones (Figure 1). This supports the hypothesis that outbreaks are typically driven by the local expansion of a single introduction event. In addition, we identified genomic links between isolates from different outbreaks and from geographically distinct regions in Germany (Additional file 1). These findings suggest the existence of underlying epidemiological connections between outbreaks, although identifying the exact transmission routes currently remains challenging.

Potential pathways of dissemination include animal population movement, i.e. wildlife and livestock, as well as trade-related introductions involving livestock or animal products. While direct evidence for these routes is limited, the observed genomic clustering calls for further epidemiological tracing and integration of genomic and field data. The results furthermore highlight the value of high-resolution genomics for outbreak investigations, i.e. additional 0-cgSNP (Additional file 1), enabling the identification of transmission networks, sources of infection, and critical control points at the animal production and wildlife interface.

Phylogenetic and spatial analyses point to both long-term persistence and geographic dissemination of highly similar *P. multocida* B:2 isolates in Germany. Clusters of near-identical isolates were detected for multiple years and across geographically distant regions (Figure 2), suggesting the presence of stable local reservoirs and/or repeated reintroductions from persisting sources. As an illustrating example, consider Subcluster 1, based on 0 cgSNP differences (Additional file 1). This genetically uniform clone persisted in the federal state of Brandenburg for over a decade, with detections spanning from 2010 to 2021. Moreover, over the course of 14 years, the same clone was also identified in five further federal states across northern and central Germany. In contrast, other clusters (e.g. 8 and 11) exhibited patterns indicative of recent spread, supporting the role of animal transport or environmental vectors in facilitating regional transmission. No host-specific clades or phylogenetic groupings were observed (Additional file 1), indicating that the evolution of *P. multocida* B:2 in Germany has not led to host-adapted lineages. This host-generalist behaviour may reflect broad ecological plasticity and highlights the pathogen's capacity to persist and spread across multiple animal species. However, to statistically confirm this hypothesis, further isolates are required.

The high number of distinct 0-cgSNP types identified is striking (Additional file 2). The coexistence of local SNP types in Mecklenburg-Western Pomerania (Cluster 4 and Subcluster 4; Cluster 7)—indicative of *P. multocida* B:2 isolates persisting in small, remote endemic foci—or widely dispersed lineages (Cluster 1; Cluster 2) present a significant challenge for effective surveillance and control. This is further complicated by the extensive and poorly defined reservoirs of *P. multocida* B:2 in wildlife. Our genomic data merely suggest that nearly identical isolates can persist over time, likely maintained in undiagnosed and/or underdiagnosed transmission chains, wildlife reservoirs, or environmental niches. Our data, at present, cannot distinguish between true clonal persistence and/or reintroduction of genetically similar strains.

It is furthermore also plausible to assume that this situation is also encountered in other countries with a history of severe HS outbreaks, e.g. in Hungary, as demonstrated by the presence of the endemic sequence type SRR1383834 [4].

The ensuing re-emergence of HS/infection with *P. multocida* B:2 (iPmB:2) and its spread across Germany was probably driven by a convergence of ecological and socio-economic factors and needs to be determined by future research. The first recognised outbreak occurred near the military training area of Altengrabow on the border of Saxony-Anhalt and Brandenburg, following a classic epidemic pattern. During the exceptionally hot summer of 2010, HS/infection with *P. multocida* B:2 (iPmB:2) appears to have spilled over from its enzootic reservoir to wild fallow deer, then to fenced game and farm animals. Owing to its high contagiousness and ability to spread via infected animals or contaminated vectors, *P. multocida* B:2 isolates from Cluster 1 (based on 10 cgSNPs) initially disseminated locally and then throughout Germany.

According to the literature outbreaks tend to subside within two to three years as host populations develop partial immunity, which is a typical feature of this pathogen. Vaccination campaigns and application of antibiotics additionally influence both the extent and duration of outbreaks [3, 23]. The presented data do not permit the final tracing of transmission events.

Similar ecological and epidemiological circumstances, together with increasing awareness of the “characteristic” clinical presentation and high mortality, facilitated the detection of further outbreaks (Additional file 1). This enabled the identification of seven circulating additional SNP clusters (2–8; based on 10-cgSNPs), which additionally increased the risk of broader dissemination. Moreover, intensified surveillance activities during diagnostic investigations—predominantly those targeting African swine fever—likely contributed to the recognition of HS/infection with *P. multocida* B:2 (iPmB:2), and future monitoring programs, e.g. screening for foot-and-mouth disease, may play a similar role.

Of note is also the clonal relationship between Hungarian and German *P. multocida* B:2 isolates indicative of a possible anthropogenic origin of transboundary outbreaks. Clonality may reflect trade relations between both countries or the presence of a third party delivering contaminated goods or animals to both countries in 2010 and 2013.

## Conclusions

The present study has (i) confirmed the limited resolution capacity of conventional MLST at distinguishing closely related *P. multocida* B:2 isolates; (ii) has shown that hierarchical clustering based on a threshold of 10 cgSNPs provides the best fit with the defined outbreak events in the German isolate collection; (iii) the 10 cgSNPs threshold needs to be independently validated in a separate dataset before being considered a universal threshold for *P. multocida* B:2. To fully elucidate the dynamics of *P. multocida* B:2, future investigations should include geographically more diverse sampling, longitudinal surveillance, and incorporation of genomic data with detailed epidemiological and ecological information. Such efforts are essential in order to differentiate between endemic persistence and exogenous introductions, and to design targeted, evidence-based interventions for managing HS/infection with *P. multocida* B:2 (iPmB:2) in both domestic and wild animal populations.

Finally, the following should also be mentioned. The absence of an efficacious vaccine not only increases animal suffering and severe economic loss for the owner, it also contributes to the spread of disease.

## Supplementary Information

**Additional file 1. SNP based dendrogram of all isolates. **Legends for metadata boxes read from left to right.

**Additional file 2. Geographic distribution of isolates across Germany.** Each location is coloured according to its 0-cgSNP cluster, visualising the spatial spread and distribution of genetically related isolates. Federal states are labelled as follows: BW, Baden-Wuerttemberg; BY, Bavaria; BB, Brandenburg; HE, Hesse; NI, Lower Saxony; MV, Mecklenburg-Western Pomerania; RP, Rhineland-Palatinate; SN, Saxony; ST, Saxony-Anhalt; TH, Thuringia.

**Additional file 3. Epidemiological metadata of the analysed German** ***Pasteurella multocida*** **B:2 isolates and results from hierarchical clustering based on SNP calling.**

**Additional file 4. Quality metrics, lipopolysaccharide (LPS) groups, and capsular (CAP) types.**

**Additional file 5. Multilocus sequence typing (MLST) using both the RIRDC and multihost schemes.**

**Additional file 6. Cluster assignments obtained using four genomic typing approaches at different clustering thresholds.**

**Additional file 7. Assembly based lipopolysaccharide (LPS) groups of European** ***Pasteurella multocida* isolates.**

## Data Availability

The datasets used and/or analysed during the current study have been submitted to ENA under the accession number PRJEB115250 (ERP195472) and will be available upon publication of the manuscript.
