# Supplementary material for: The genomic landscape of Pasteurella multocida B:2 causing haemorrhagic septicaemia in Germany
Source: Vet Res. 2026 Oct 29;57:188. doi: 10.1186/s13567-026-01868-x (PMC13721223; doi:10.1186/s13567-026-01868-x)
Supplement: Supplementary file 1 [file 13567_2026_1868_MOESM1_ESM.pdf]

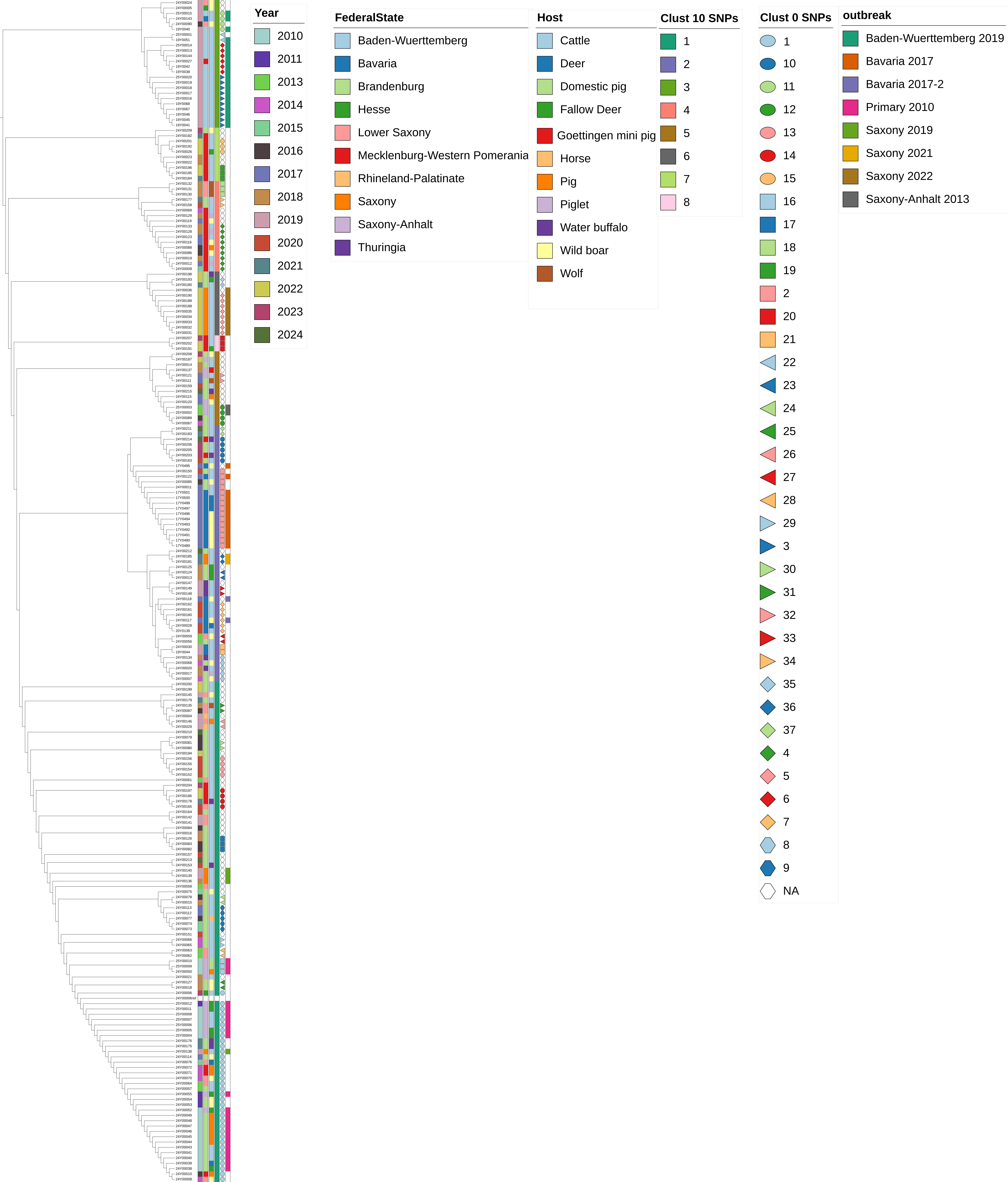
Year
2010
2011
2013
2014
2015
2016
2017
2018
2019
2020
2021
2022
2023
2024
FederalState
Baden-Wuerttemberg
Bavaria
Brandenburg
Hesse
Lower Saxony
Mecklenburg-Western Pomerania
Rhineland-Palatinate
Saxony
Saxony-Anhalt
Thuringia
Host
Cattle
Deer
Domestic pig
Fallow Deer
Goettingen mini pig
Horse
Pig
Piglet
Water buffalo
Wild boar
Wolf
Clust 10 SNPs
1
2
3
4
5
6
7
8
Clust 0 SNPs
1
10
11
12
13
14
15
16
17
18
19
2
20
21
22
23
24
25
26
27
28
29
3
30
31
32
33
34
35
36
37
4
5
6
7
8
9
NA
outbreak
Baden-Wuerttemberg 2019
Bavaria 2017
Bavaria 2017-2
Primary 2010
Saxony 2019
Saxony 2021
Saxony 2022
Saxony-Anhalt 2013
