# Supplementary material for: The genomic landscape of Pasteurella multocida B:2 causing haemorrhagic septicaemia in Germany
Source: Vet Res. 2026 Oct 29;57:188. doi: 10.1186/s13567-026-01868-x (PMC13721223; doi:10.1186/s13567-026-01868-x)

Clustering (0 cgSNPs)

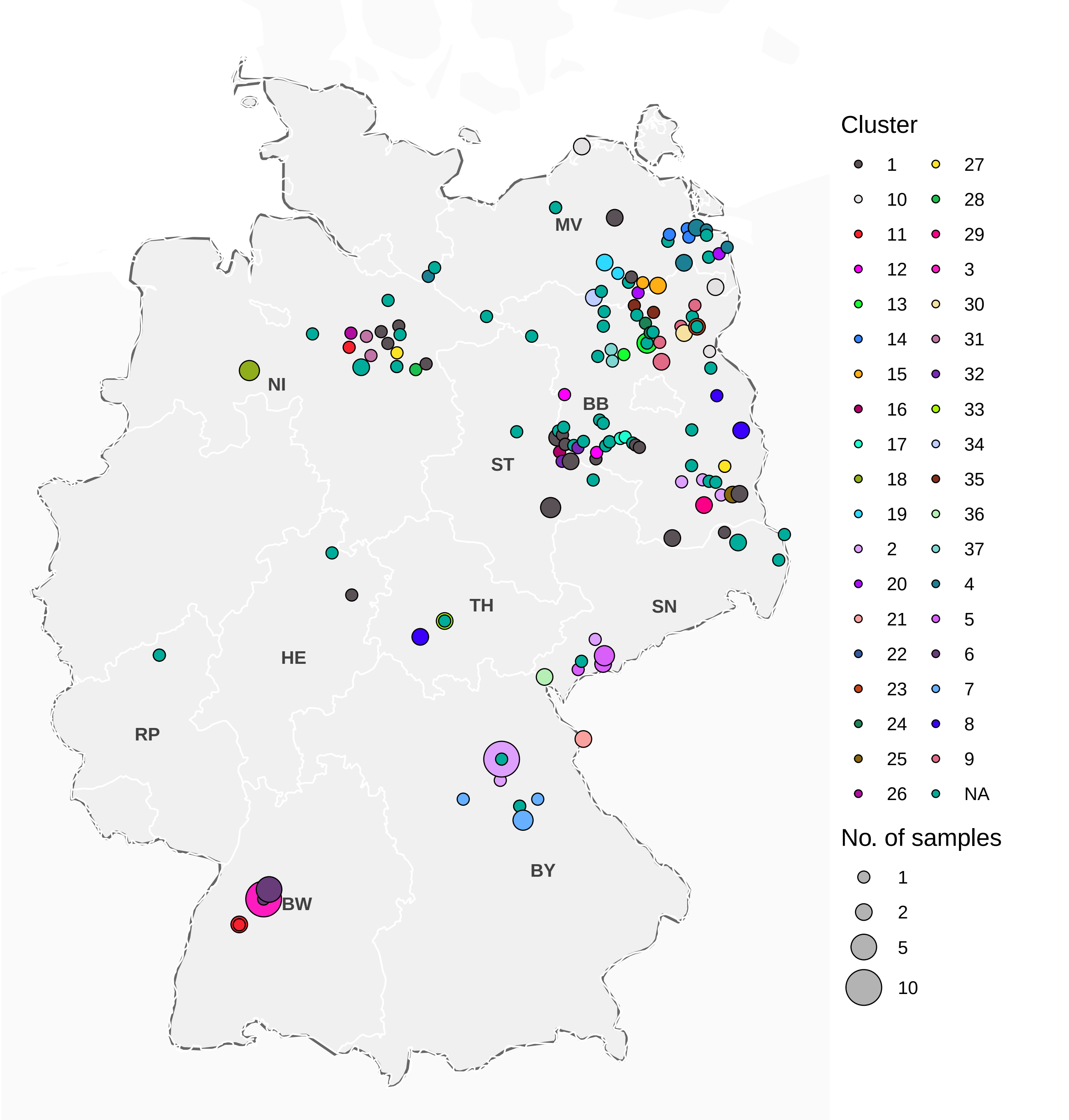

Supplement: Supplementary file 2 [file 13567_2026_1868_MOESM2_ESM.pdf]
